# Anomaly Detection of Power Plant Equipment Using Long Short-Term Memory Based Autoencoder Neural Network

**DOI:** 10.3390/s20216164

**Published:** 2020-10-29

**Authors:** Di Hu, Chen Zhang, Tao Yang, Gang Chen

**Affiliations:** School of Energy and Power Engineering, Huazhong University of Science and Technology, Wuhan 430074, China; hudi1994@hust.edu.cn (D.H.); zhangchen710@yeah.net (C.Z.); gangchen@hust.edu.cn (G.C.)

**Keywords:** anomaly detection, power plant, artificial neural networks, long short-term memory based autoencoder neural networks, normal behavior model

## Abstract

Anomaly detection is of great significance in condition-based maintenance of power plant equipment. The conventional fixed threshold detection method is not able to perform early detection of equipment abnormalities. In this study, a general anomaly detection framework based on a long short-term memory-based autoencoder (LSTM-AE) network is proposed. A normal behavior model (NBM) is established to learn the normal behavior patterns of the operating variables of the equipment in space and time. Based on the similarity analysis between the NBM output distribution and the corresponding measurement distribution, the Mahalanobis distance (MD) is used to describe the overall residual (OR) of the model. The reasonable range is obtained using kernel density estimation (KDE) with a 99% confidence interval, and the OR is monitored to detect abnormalities in real-time. An induced draft fan is chosen as a case study. Results show that the established NBM has excellent accuracy and generalizability, with average root mean square errors of 0.026 and 0.035 for the training and test data, respectively, and average mean absolute percentage errors of 0.027%. Moreover, the abnormal operation case shows that the proposed framework can be effectively used for the early detection of abnormalities.

## 1. Introduction

In recent years, attention has been devoted to the improvement of condition monitoring systems (CMSs) for power plants [1,2]. One of the critical tasks is anomaly detection for equipment, which can be used to reduce the high cost of unplanned downtime and has great significance in condition-based maintenance. Anomaly detection refers to the detection of patterns that do not conform to established normal behavior models (NBMs) in a specified dataset. The patterns detected are called anomalies [3]. The CMSs store a large amount of operation data in power plants [4,5,6], and the use of this data represents an important field for future smart power plants (SPPs) [7,8]. Therefore, anomaly detection using data-driven approaches is a popular research topic for SPPs. Power plant equipment can be regarded as a dynamic system with *n* operating variables, and NBMs describe the normal behavior patterns among these variables. The data-driven modeling approach establishes NBMs based on the historical normal operation dataset, without knowledge of the precise mechanism of the system. The residuals between the NBM outputs and measured variables are monitored in real-time. When the residual exceeds the threshold, the system is considered to be abnormal [9].

Machine learning methods were used to create NBMs as early as 20 years ago [10,11,12], due to their ability to model nonlinear dynamic systems. However, early computing power was limited, and the structure of NBMs was very simple, and included one output. Following the development of hardware and machine learning methods, many anomaly detection methods based on artificial neural networks (ANNs) and support vector machines (SVMs) have been proposed, e.g., back propagation neural network (BPNN) [13,14,15], SVM [16,17], least squares support vector machine (LS-SVM) [18,19], and restricted Boltzmann machines (RBM) [20]. However, these methods do not consider the time sequence correlation between variables. The research of Safdarnejad et al. [21] showed that dynamic model considering the time-series data has higher prediction accuracy. The nonlinear autoregressive neural network with exogenous input (NARX) model considers the time-series relationship by time lag or tapped delay line (TDL) [22,23,24,25]. However, the time lag increases the input layer dimensions, which in turn increases the risks of over-fitting. Moreover, the time lag must be manually set. Recurrent neural networks (RNNs) are widely used in time-series modeling [26,27,28,29]. However, the conventional RNN still requires the model’s input variables to be manually selected according to the output variables. Equipment operation represents a multivariable time-series that requires an NBM to have multiple outputs. In addition, according to the output of NBM, there is no clear criterion for the selection of input variables. Monitoring multiple variables using multiple models is time-consuming and costly. Therefore, it is necessary to find a method that can monitor multiple variables simultaneously and does not require a complicated variable selection process. The autoencoder (AE) neural network is an unsupervised learning model, whose output is the reconstruction of the input, thus avoiding manual selection of feature variables. However, the conventional AE-based methods also do not consider the time sequence correlation between variables in power plants, as previously discussed, e.g., AE [30,31], stacked autoencoder (SAE) [32,33], variational autoencoder (VAE) [34], and deep autoencoder Gaussian mixture model [35].

In this study, the long short-term memory-based autoencoder (LSTM-AE) neural network is proposed to establish an NBM with multiple outputs, which learns the correlation between variables in time and space. The advantages of the LSTM-AE are as follows: Firstly, the autoencoder (AE) does not need to manually select the inputs according to the outputs. Secondly, the long and short-term memory (LSTM) can learn the relationship of the variables in a time-series and reduce the long-term dependence. A general anomaly detection framework for power plant equipment is proposed. Based on the similarity analysis between the NBM output distribution and the corresponding measurement distribution, the Mahalanobis distance (MD) is used to describe the overall residual (OR) of the model. The reasonable range of residuals is obtained using kernel density estimation (KDE) with a 99% confidence interval. Taking an induced draft fan as an example, the NBM was established with eight output variables. Hyperparameters such as look-back time steps, and the number of hidden layer units, were optimized. The model accuracy is compared with previous work. Furthermore, the process of anomaly detection is demonstrated via a case study.

The main contributions of this study are in two parts:

1. The proposal of an anomaly detection framework for multivariable time-series based on an LSTM-AE neural network.
2. The similarity analysis of the NBM output distribution and the corresponding measurement distribution.

The remainder of this paper is organized as follows: In Section 2, the proposed anomaly detection framework based on the LSTM-AE neural network is presented in detail. In Section 3, the case study demonstrates the established NBM, comparative analysis, and the process of anomaly detection in a power plant. The conclusions and future work are provided in Section 4.

## 2. The Proposed Anomaly Detection Framework

### 2.1. Framework Flow Chart

**Figure 1:**
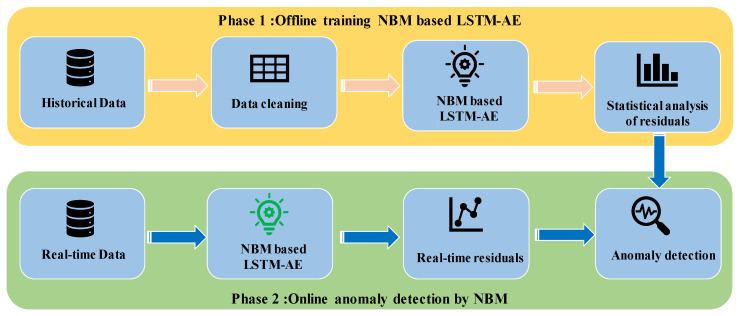
Anomaly detection framework based on the long short-term memory-based autoencoder (LSTM-AE) neural network.

Diverse pieces of equipment are utilized in a power plant. In this paper, a general anomaly detection framework for power plants is proposed. The framework utilizes a large amount of operating data relating to the equipment and adopts the LSTM-AE network to establish the NBM. The residual is calculated using the NBM outputs and corresponding measured variables. The residual threshold is obtained using residual statistical analysis of the test dataset. The proposed anomaly detection framework includes two phases, as shown in Figure 1.

#### 2.1.1. Offline Training NBM

The purpose of this phase is to establish the NBM of the target equipment and obtain a reasonable range of model residuals. Specific steps are as follows:

Step 1: According to the target equipment, obtain the historical dataset of related operating variables.

Step 2: Performing data cleaning to obtain the normal behavior dataset based on three aspects:

(1) Eliminate data at the time of equipment downtime.
(2) Eliminate data at the time of equipment failure according to operation logs.
(3) Eliminate abnormal data based on statistical characteristics. These abnormalities originate from sensors or stored procedures. Boxplots are used in this work. Then, the cleaned dataset is divided into a training dataset and a test dataset.

Step 3: The NBM is established based on an LSTM-AE with the training dataset.

Step 4: Statistical analysis of the residuals with the test dataset is performed. The MD is proposed to describe the OR of multiple variables, and the reasonable range of residuals is obtained using KDE with a 99% confidence interval.

#### 2.1.2. Online Anomaly Detection by NBM

The purpose of this phase is to perform real-time anomaly detection on the target equipment based on the trained NBM. Specific steps are as follows:

Step 1: Based on the trained NBM and measured variable values, the residuals are generated in real-time to reflect the status of the equipment.

Step 2: Based on the real-time OR of the model and the residual of each variable, the abnormal patterns are detected through the reasonable range. This is referred to as phase 1.

### 2.2. The Normal Behavior Model

The NBM can represent the dynamic relationships among variables in a system [9]. When the system is normal, the output value of the NBM is consistent with the measured value. When the system is abnormal, the output value is different from the measured value. In this study, the LSTM-AE neural network is proposed to establish NBM. Suppose that certain equipment has *n* operating variables, $x^{n}$ represents the measured values at a certain moment, and ${\hat{x}}^{n}$ represents the reconstruction of $x^{n}$. The residuals of $x^{n}$ and ${\hat{x}}^{n}$ are used to judge whether the system is abnormal. The threshold α is determined by KDE of the reconstruction residuals with a 99% confidence interval. The NBM is trained with a normal operation dataset of the equipment. Algorithm 1 shows the anomaly detection using the NBM.


**Algorithm 1 Anomaly detection using the NBM**
**INPUT**: normal dataset X, the measured values at a certain moment $x^{n}$, threshold α**OUTPUT**: reconstruction residual $||x^{n}-\;{\hat{x}}^{n}||\;$  f(·) represents the NBM trained by X
  **if** reconstruction residual > α
**then**    $x^{n}$ is an anomaly  **Else**    $x^{n}$ is not an anomaly  
end


### 2.3. The LSTM-AE Neural Network

#### 2.3.1. The AE Neural Network

**Figure 2:**
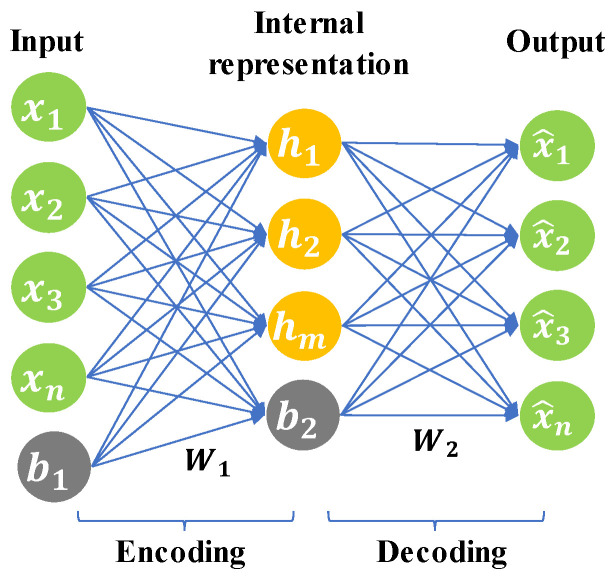
The structure of the autoencoder (AE) neural network.

The AE neural network is an unsupervised ANN with a hidden layer [36]. It has a three-layer symmetrical structure, as shown in Figure 2, including an input layer, a hidden layer (interval representation), and an output layer (reconstruction). The input layer to the hidden layer is the encoding process, and the hidden layer to the output layer is the decoding process. The goal of the AE is to reconstruct the original input as much as possible.

The encoding process:

(1) $$H=f_{1}\left(W_{1}\;\cdot X\;+{\;b}_{1}\right)$$

The decoding process:

(2) $$\hat{X}=f_{2}\left(W_{2}\;\cdot H\;+\;b_{2}\right)$$

where $W_{1}$ and $b_{1}$ represent the weight and bias from the input layer to the hidden layer, respectively. $W_{2}$ and $b_{2}$ represent the weight and bias from the hidden layer to the output layer, respectively. X, H, and $\hat{X}$ represent the original input, intermediate representation, and reconstruction of the original data, respectively. $f_{1}()$ and $f_{2}()$ are activation functions. Common activation functions are the sigmoid function, tanh function, and ReLu function [37].

The most important feature of the AE is that the decoded $\hat{X}$ should be close to the original input X to the greatest extent; that is, the residual between $\hat{X}$ and X needs to be minimized. Therefore, the reconstruction error can be calculated as follows:

(3) $$J\left(W,b\right)=\;\overset{N}{\underset{i=1}{\sum }}\overset{M}{\underset{j=1}{\sum }}||{\hat{x}}_{ij}-\;x_{ij}||{}^{2}$$

where N and M represent the dimensions of the original data and the number of samples, respectively. The goal of model training is to find $W\left(W_{1},\;W_{2}\right)$ and $b\left(b_{1},\;b_{2}\right)$ that minimize the loss function.

#### 2.3.2. The LSTM Unit

**Figure 3:**
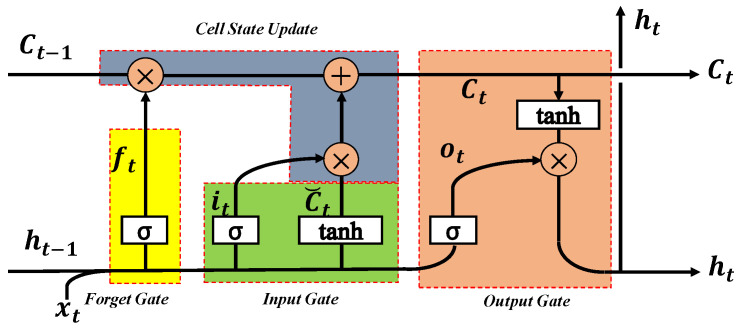
Architecture of a long short-term memory (LSTM) unit.

The RNN uses internal state (memory) to process the time-series input to capture the relationship of input variables in sequence. The backpropagation through time (BPTT) algorithm is typically employed to train the RNN [38,39,40]. However, the chain derivation rule of the BPTT training algorithm results in gradient vanishing or explosion problems for long-term dependency tasks. The LSTM unit combines short-term memory with long-term memory using subtle gate control and solves the problem of gradient disappearance to a certain extent [41], as illustrated in Figure 3.

The pre-built memory unit consists of three gate structures: an input gate, a forget gate, and an output gate. The input and output gates control input and output activation to the memory cell, respectively, whereas the forget gate updates the state of the cell. These gate structures constitute a self-connected constant error conveyor belt (CEC), which allows the constant error flow to pass through the internal state to solve the problem of gradient disappearance. The memory cells are updated and the output is implemented by the following equations [41]:

(4) $$f_{t}=sgmoid\left(W_{xf}\cdot x_{t}+\;W_{hf}\cdot h_{t-1}+b_{f}\right)$$

(5) $$i_{t}=sgmoid\left(W_{xi}\cdot x_{t}+\;W_{hi}\cdot h_{t-1}+b_{i}\right)$$

(6) $${\overset{˘}{C}}_{t}=tanh\left(W_{xa}\cdot x_{t}+\;W_{ha}\cdot h_{t-1}+b_{a}\right)$$

(7) $$o_{t}=sgmoid\left(W_{xo}\cdot x_{t}+\;W_{ho}\cdot h_{t-1}+b_{o}\right)$$

(8) $$C_{t}=f_{t}⊗C_{t-1}+\;i_{t}⊗{\overset{˘}{C}}_{t}$$

(9) $$h_{t}=o_{t}⊗tanh\left(C_{t}\right)$$

where $h_{t-1}$ and $C_{t-1}$ are output and cell state at the previous moment, respectively; $x_{t}$ represents the current input; f represents the forget gate; $f_{t}$ represents the forgetting control signal of the cell state at the previous moment; $f_{t}⊗C_{t-1}$ represents the information retained at the previous moment; i represents the input gate; ${\overset{˘}{C}}_{t}$ represents the candidate cell state at the current moment; $i_{t}$ represents the control signal for ${\overset{˘}{C}}_{t}$; o represents the output gate; $h_{t}$ represents the final output; $o_{t}$ represents the output control signal; $W_{x}$ represents the input-hidden layer connection matrix; $W_{h}$ indicates the hidden layer recurrent connection matrix; ⊗ represents elementwise multiplication.

#### 2.3.3. The LSTM-AE Neural Network

Abnormal detection of power plant equipment requires simultaneous monitoring of multiple variables. An AE-based NBM can reconstruct multiple variables at the same time without manually selecting input variables. These variables in power plant equipment are not only spatially correlated, but also have a strong temporal correlation. The conventional AE neural network cannot mine the time correlation of input layer variables.

**Figure 4:**
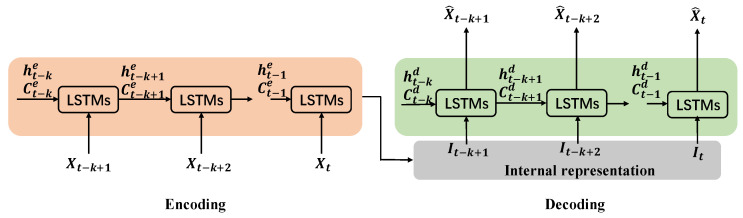
Architecture of the LSTM-AE.

For this purpose, the LSTM-AE neural network is proposed with the hidden layer units replaced by LSTM units. The LSTM-AE neural network cannot only learn the correlation between input variables, but also learn the correlation in the time series. At the same time, the LSTM unit can avoid the problem of long-term memory dependence. The structure of the LSTM-AE is depicted in Figure 4.

In Figure 4, X is the origin input; $\hat{X}$ is the output, reconstruction of X; I is the encoding result; k represents the look-back time steps in the LSTM unit; $h^{e}$ and $C^{e}$ are the output and cell state, respectively, of the LSTM unit in the encoding process; $h^{d}$ and $C^{d}$ are the output and cell state, respectively, of the LSTM unit in the decoding process.

## 3. Case Study

### 3.1. Data Preparation

In this study, an induced draft fan is considered as an example, with a historical dataset of the related temperature variables obtained from the CMS. The obtained dataset is from 1 May 2019 to 1 May 2020, with a 1 min interval. Eight temperature variables are contained in the dataset, as follows:

Lubricating oil temperature: Abbreviated as LOT. Lubricating oil is used to cool the bearings in the induced draft fan.

Outlet temperature of electrostatic precipitator A1: Abbreviated as POT_A1, and indicates the temperature of the flue gas from the electrostatic precipitator A1 into the induced draft fan.

Outlet temperature of electrostatic precipitator A2: Abbreviated as POT_A2, and indicates the temperature of the flue gas from the electrostatic precipitator A2 into the induced draft fan.

Bearing temperature at the non-drive end of the motor: Abbreviated as MNDT, and indicates the health of the bearing.

Bearing temperature at the drive end of the motor: Abbreviated as MDT, and indicates the health of the bearing.

Main bearing temperature 1: Abbreviated as MBT_1, and reflects the health status of the bearing.

Main bearing temperature 2: Abbreviated as MBT_2, and reflects the health status of the bearing.

Main bearing temperature 3: Abbreviated as MBT_3, and reflects the health status of the bearing.

**Figure 5:**
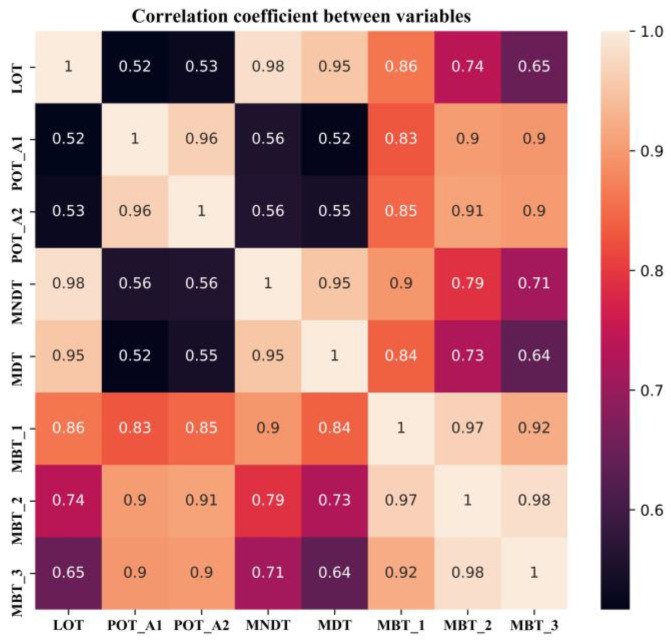
The correlation coefficient between variables.

The correlation coefficient matrix indicates the correlation between the variables; as shown in Figure 5, there is a clear correlation between the variables.

### 3.2. Data Cleaning

**Figure 6:**
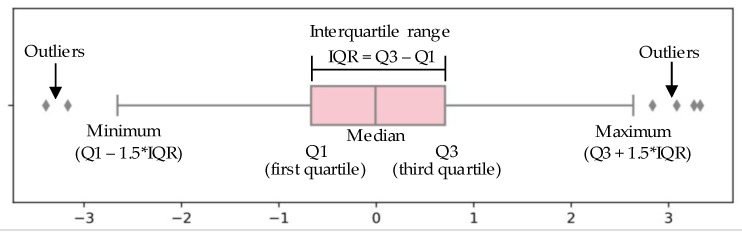
Boxplot outlier detection principle.

**Figure 7:**
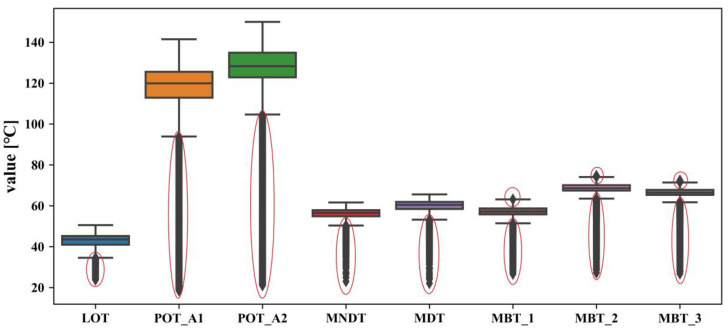
Data cleaning by boxplot.

In principle, the normal behavior dataset is required to establish an NBM. It is necessary to clean the acquired historical data set. Firstly, the dataset is cleaned according to the equipment operating signal. Furthermore, the data during the failure period is eliminated according to the operation log. In this study, the boxplot method is used to eliminate abnormal data caused by sensors and the stored procedure from the perspective of statistics. A boxplot is a standardized way of displaying the dataset based on a five-number summary: the minimum, the maximum, the sample median, and the first and third quartiles. When the data exceeds the minimum and maximum range, it is considered an outlier [42]. The principle of outlier detection using a boxplot is shown in Figure 6. The data cleaning results in this study are shown in Figure 7, and the points in red circles are considered outliers that will be eliminated.

### 3.3. NBM Based on LSTM-AE

In this study, the NBM of an induced draft fan was established using an LSTM-AE neural network with eight temperature variables as model inputs. After data cleaning, 266,538 observations were obtained in the sample. Fifty percent of the sample comprised the training dataset, and the remaining 50% comprised the test dataset. Min-max normalization was adopted, and the range of variables was scaled to [0,1]. For the evaluation of the developed NBM model of the induced draft fan, two indices— root mean square error (RMSE) and mean absolute percentage error (MAPE)—were used. The definitions of the two indices are given in the following equations:

(10) $$RMSE=\;\sqrt{\frac{1}{n}\overset{n}{\underset{i=1}{\sum }}{\left({\hat{y}}_{i}-y_{i}\right)}^{2}}$$

(11) $$MAPE=\frac{1}{n}\overset{n}{\underset{i=1}{\sum }}\left|\frac{{\hat{y}}_{i}-y_{i}}{y_{i}}\right|\times 100\%$$

where $y_{i}$ is measured value, ${\hat{y}}_{i}$ is reconstructed value, and n is the number of variables.

**Figure 8:**
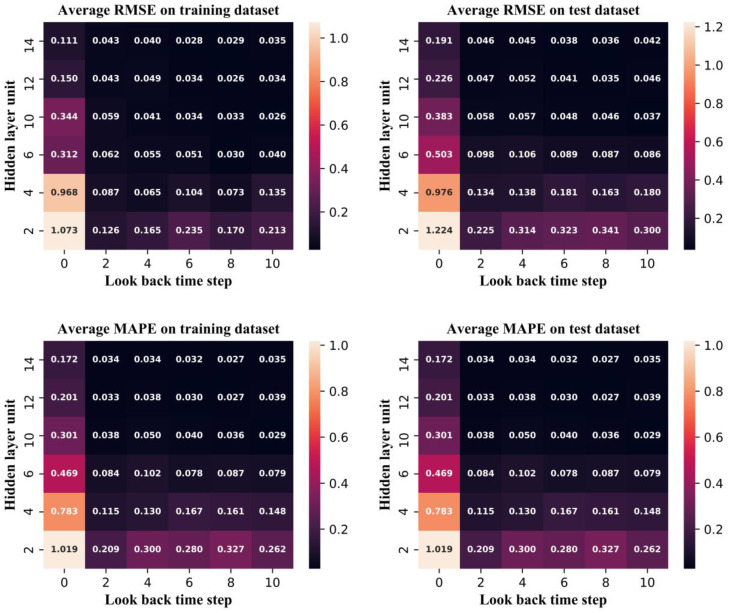
The performance of the LSTM-AE model on difference combination.

The look-back time step and hidden layer unit are important hyperparameters in the LSTM-AE and determine the structure of the NBM. Many different combinations were tested in this work, e.g., “4–6” means that the look-back time step is 4 and the number of hidden layer unit is 6. When the look-back time step is 0, the structure is a conventional AE. The performances of different combinations as measured by the two evaluation indices are shown in Figure 8. The comparison between the LSTM-AE and AE is shown in Table 1, and indicates that LSTM-AE is significantly better.

It can be clearly seen from Figure 8 that when the number of hidden layer units is small, the model performance is more sensitive to the number of hidden layer units than the look-back time step; however, a small look-back time step can also effectively improve the model performance. Therefore, the time relationship between variables is meaningful for improving model performance. However, when the number of hidden layer elements is very small, an overly large look-back time step will lead to the degradation of model performance. When the look-back time step exceeds 0 and the number of hidden layer units is greater than six, the RMSE and MAPE of the training and test datasets are highly similar and very low, which indicates that the accuracy and generalization of the model are very high. However, increasing the look-back time steps and the number of hidden layer units leads to an increase in the required model training resources. In this study, while ensuring the accuracy of the model, a small-scale model structure should be selected as far as possible; thus, the combination “8–12” was selected as the final model structure. 

#### Comparative Analysis

In previous research, the principle components analysis- nonlinear autoregressive neural network with exogenous input (PCA-NARX) method was proposed to establish the NBM [25]. PCA was used to reduce redundancy and retain useful information of dataset. The NARX is a nonlinear autoregressive model which has exogenous inputs and can be stated algebraically as:

(12) $$y_{t}=F\left(y_{t-1},\;y_{t-2},\ldots ,y_{t-n_{y}},\;u_{t-1},u_{t-2\;},\;\ldots ,u_{t-n_{u}}\right)+\;\varepsilon_{t}$$

where y is the variable of interest; u is the exogenous variable; $n_{y}$ is the TDL (or time lags) of y, which indicates that previous values help to predict y; $n_{u}$ is the TDL of u; ε is the error term; F() is the neural network. In this model, information about u helps predict y, as do previous values of y itself.

**Figure 9:**
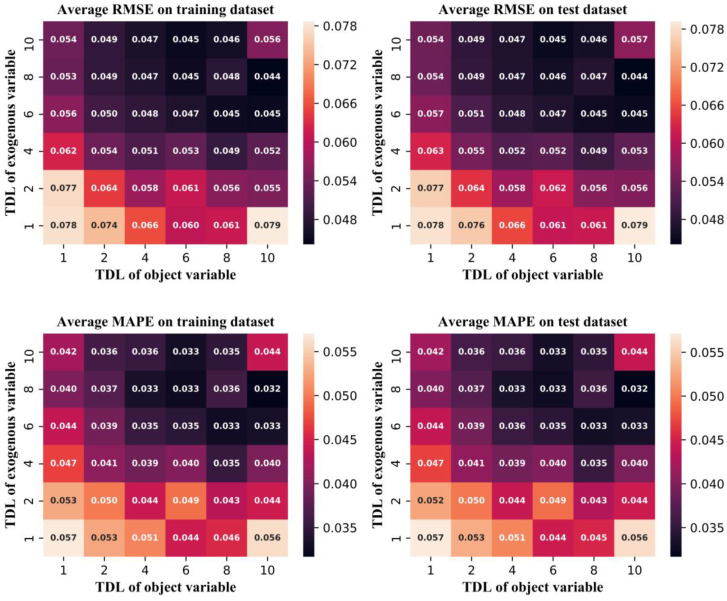
The performance of the principle components analysis- nonlinear autoregressive neural network with exogenous input (PCA-NARX) model for different combinations.

In the PCA-NARX method, the exogenous variable is the PCA result of the object variable. In this study, an NBM based on PCA-NARX was established. The TDL of the object and exogenous variables were hyperparameters, and a numerous combinations were trained. The performance of PCA-NARX for different combinations is shown in Figure 9. The comparison of the best performance between the LSTM-AE and PCA-NARX is shown in Table 2. The PCA-NARX method considers the time sequence correlation between variables, so is better than the conventional AE method. However, LSTM-AE is better than PCA-NARX. From the perspective of model principles, PCA-NARX is a predictive model, and LSTM-AE is a reconstruction model.

### 3.4. Statistical Analysis on the Residuals

In this study, a total of 133,405 observations were obtained from the corresponding reconstruction by the trained NBM. The MD was proposed to describe the overall residual (OR) of NBM. The MD provides the univariate distance between multi-dimensional samples that obey the same distribution with consideration of the correlation between variables and the dimensional scale, and has been successfully applied to capture different types of outliers [43]. The MD can be calculated as follows:

(13) $$MD_{ij}=\;\sqrt{\left(X_{i}-X_{j}\right)C^{-1}{\left(X_{i}-X_{j}\right)}^{T}}$$

where $X_{i}$ and $X_{j}$ are different samples in the same distribution, and C is the covariance matrix between the variables of the distribution.

The distribution similarity between the measured dataset and the reconstructed dataset was used in this study. Kullback–Leibler (KL) divergence [44] was used to describe the distribution difference of the measurement dataset and the reconstructed dataset. KL divergence is an asymmetric and non-negative measure of the difference between two probability distributions. A smaller KL divergence demonstrates a greater similarity of two distributions. KL divergence can be calculated as depicted:

(14) $$D_{KL}(P||Q)=\;\overset{N}{\underset{i=1}{\sum }}p\left(x_{i}\right)\log\left(\frac{p\left(x_{i}\right)}{q\left(x_{i}\right)}\right)$$

where P and Q are two distributions, $p\left(x_{i}\right)$ and $q\left(x_{i}\right)$ represent the probability of the two distributions for sample $x_{i}$, respectively; N is the number of samples.

**Figure 10:**
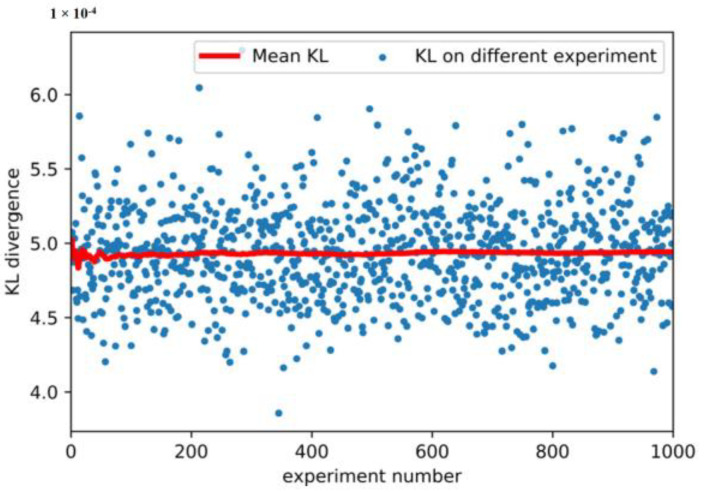
Kullback–Leibler (KL) divergence of the measured dataset and the reconstructed dataset in tests.

Because the dataset has eight dimensions and each dimension takes 100 points uniformly, a total of 10^16^ sample points exists, which causes calculation difficulties. In this work, 2000 observations were randomly selected each time, and 1000 experiments were performed. KL divergence of the measurement dataset and the reconstructed dataset with 1000 experiments is shown in Figure 10. The calculated KL divergences are very small and consistent. The red line indicates the average KL divergence change trend as the number of experiments increases. The final KL was calculated to be 0.0049 using the law of large numbers. The experimental results show that the distributions of the measurement dataset and reconstructed dataset are approximately equal; therefore, it can be considered that the reconstructed dataset and the measurement dataset belong to the same distribution. The MD was applied to describe the OR of model, and the calculation method is as follows:

(15) $$MD_{Residual}^{i}=\sqrt{\left(Y_{i}-{\hat{Y}}_{i}\right)C^{-1}{\left(Y_{i}-{\hat{Y}}_{i}\right)}^{T}}$$

where $Y_{i}$ and ${\hat{Y}}_{i}$ represent the measurement value and reconstructed value of the operating variables at time i, respectively; C is the covariance matrix between the operating variables.

The residual of each variable and overall model were subjected to statistical analysis. The KDE method was used to simulate the probability density function (PDF) of the residual distributions, to obtain a reasonable range of residuals. The KDE uses a smooth peak function to fit the observed data points to simulate the true probability distribution curve. Let $x_{1}$,$\;x_{2}$,…,$\;x_{n}$ be an independent and identically distributed data sample, then, the kernel density of its PDF is estimated as:

(16) $${\hat{f}}_{h}\left(x\right)=\frac{1}{nh}\overset{n}{\underset{i=1}{\sum }}K\left(\frac{x-x_{i}}{h}\right)$$

where h is a smoothing parameter called the bandwidth. The larger the bandwidth, the smaller the proportion of the observed data points in the final curve shape, and the flatter the overall curve; the smaller the bandwidth, the greater the proportion of the observed data points in the final curve shape. The overall curve is steeper. K(·) is the kernel function; commonly used kernel functions are triangle, rectangle, Epanechnikov, Gaussian, etc. 

**Figure 11:**
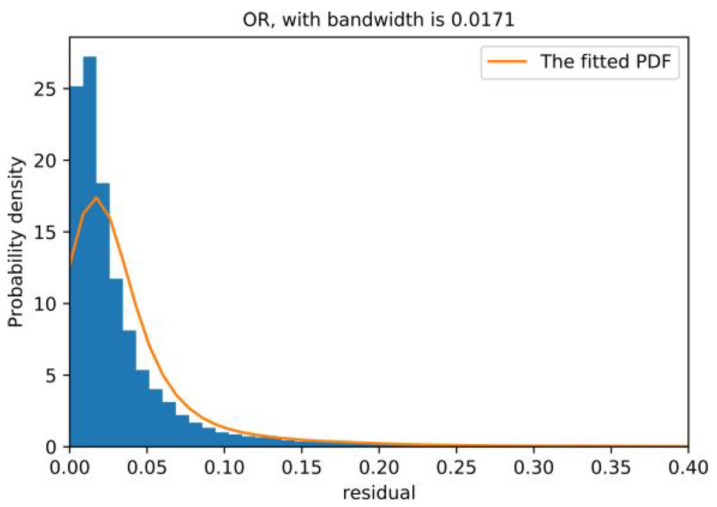
OR distribution of the normal behavior model (NBM).

**Figure 12:**
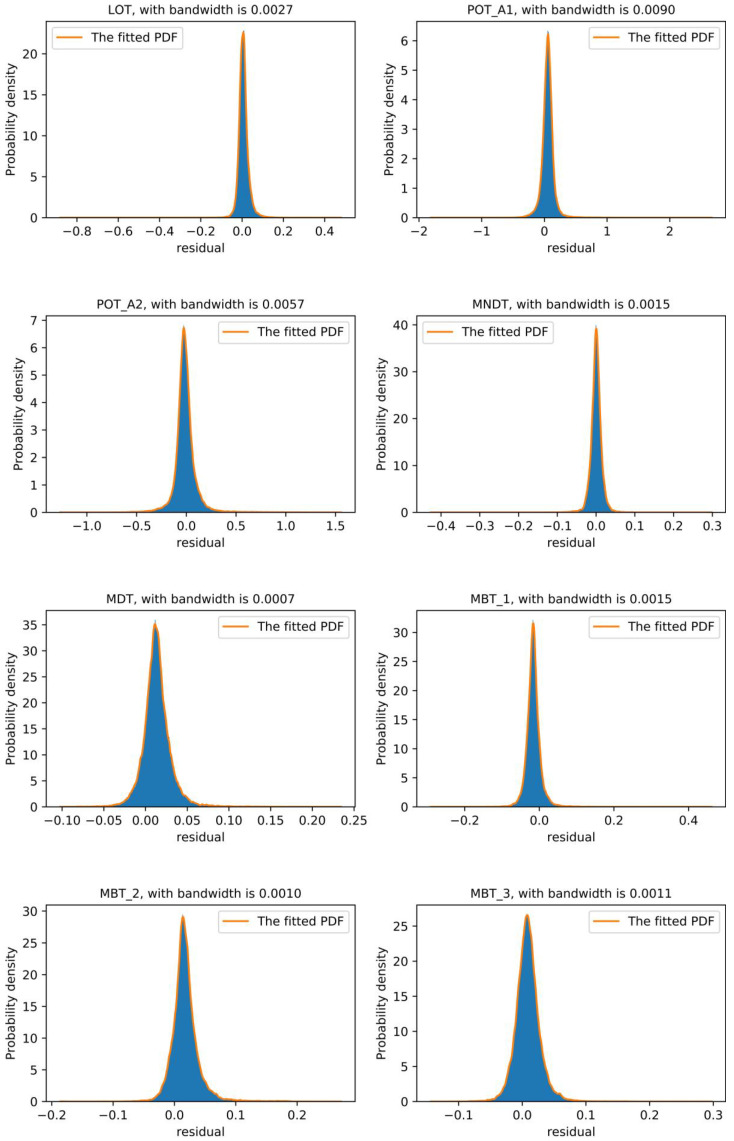
Residual distribution of each variable.

In this embodiment, the kernel function was a Gaussian function, and an adaptive bandwidth method [45] was adopted. The residual distributions of the overall NBM and each variable are shown in Figure 11 and Figure 12, respectively. F(·) represents the cumulative probability function (CDF), the integral of the probability density function. The lower and upper threshold of the reasonable residual range are recorded as R_lower and R_upper, respectively. In this work, the reasonable residual range was obtained based on the 99% confidence interval. This means F(R_lower)=0.01, F(R_upper)=0.99; the reasonable residual range of the overall NBM and each variable are shown in Table 3. The overall residual of the model only takes the upper limit.

### 3.5. Abnormality Detection

Based on the trained NBM of the induced draft fan as described in the previous section, this work demonstrates the proposed abnormality detection methods using two cases: normal operation and abnormal operation.

#### 3.5.1. Normal Operation Case

**Figure 13:**
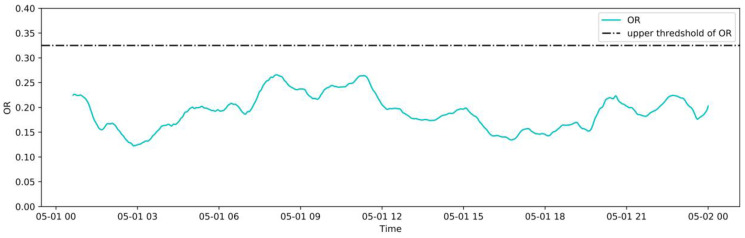
Real-time monitoring of the OR in a normal case.

**Figure 14:**
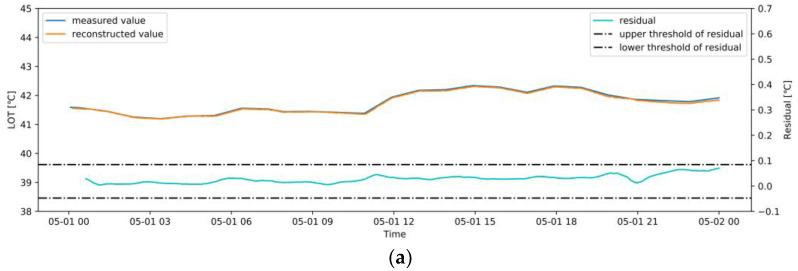

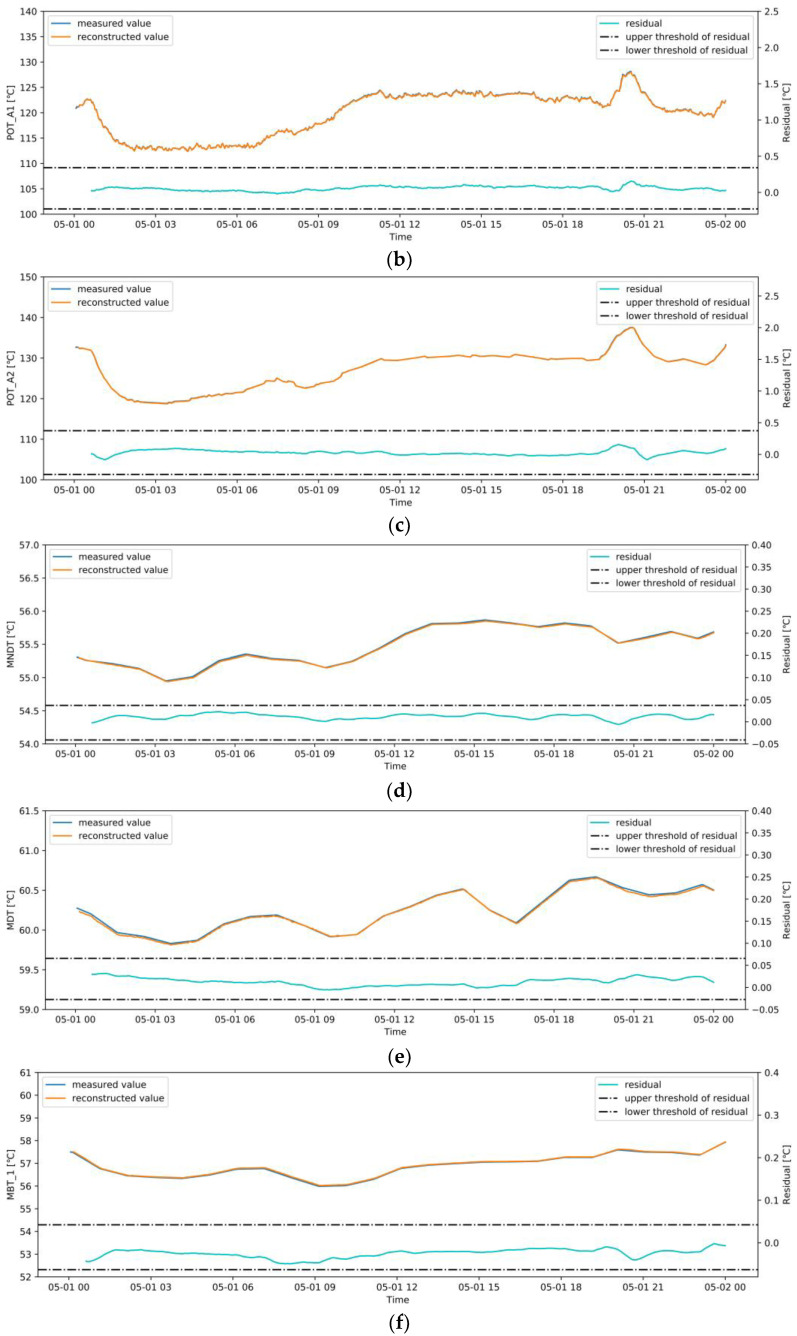

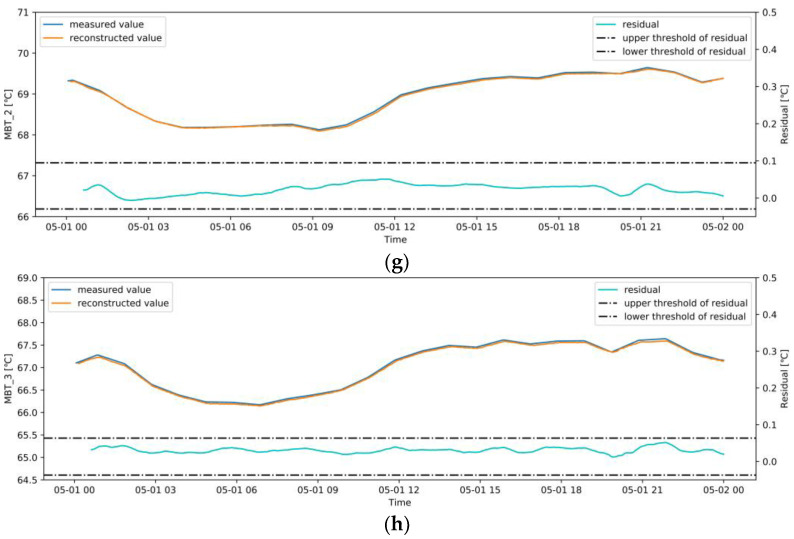
Real-time monitoring of variables in a normal case: (**a**) LOT; (**b**) POT_A1; (**c**) POT_A2; (**d**) MNDT; (**e**) MDT; (**f**) MBT_1; (**g**) MBT_2; (**h**) MBT_3.

A two-day operation dataset was collected from May 1 to May 2, 2020 with an interval of 1 min to monitor abnormalities in real-time. The real-time OR and each variable monitoring process are shown in Figure 13 and Figure 14, respectively. The measured value obtained from the CMS and the reconstructed value given by the NBM are monitored in real-time. The reasonable residual ranges of each variable are shown in Table 2. The time lag and noise in the actual operation cause a small amount of discontinuity outside the allowable residual range in the real-time monitoring process. Therefore, in the actual monitoring process, an average sliding window, with size = 30, is used to process the monitored residuals. It can be seen from Figure 13 that the OR of the model is within the reasonable range during this period, indicating that the induced draft fan operates normally and is consistent with this case. Figure 14 shows that the residual of each variable does not exceed the reasonable range, and the reconstructed values are very close to the measured values. This indicates that the trained NBM has a good generalization and accuracy.

#### 3.5.2. Abnormal Operation Case

**Figure 15:**
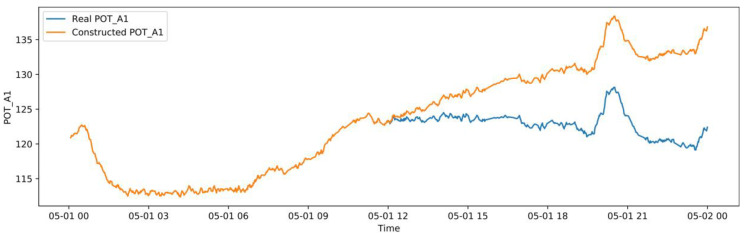
Constructed POT_A1 with linear cumulative drift.

**Figure 16:**
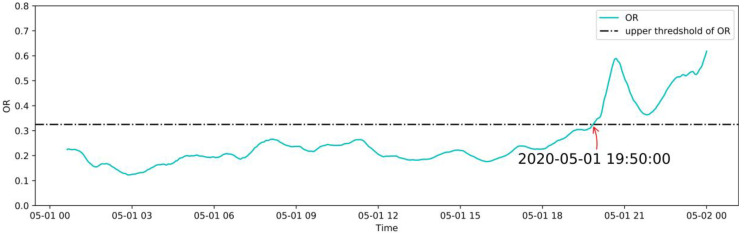
Real-time monitoring of the OR in an abnormal case.

**Figure 17:**
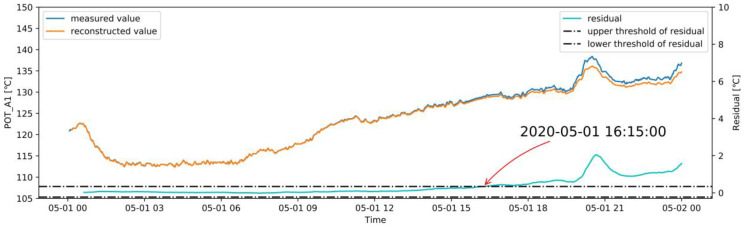
Real-time monitoring of POT_A1 in an abnormal case.

In this study, the abnormal dataset was manually constructed according to the correlation of the variables shown in Figure 5 for an early warning demonstration, because there was no real failure case of the induced draft fan. The correlation coefficients among LOT, MNDT, MDT, MBT_1, MBT_2, and MBT_3 are relatively high, and the failure data between them has high uncertainty. The abnormal dataset generation is performed using POT_A1, and the other variables remain unchanged. Based on the normal operation case, starting at 12:00 on May 1, a linear cumulative drift was added to POT_A1 with a coefficient of 0.02 °C, as shown in Figure 15. The real-time monitoring process of the OR is shown in Figure 16, which shows the OR exceeds the upper threshold at 19:50 when the cumulative drift of POT_A1 was 9.4 °C. The real-time monitoring of POT_A1 is shown in Figure 17, which shows that the residual of POT_A1 exceeds the upper threshold at 16:15 when the cumulative drift of POT_A1 is 5.1 °C. According to the conventional fixed threshold, the alarm will not be raised until POT_A1 reaches 180 °C. In this case, the OR exceeds the threshold when POT_A1 is 132.23 °C, and the residual of POT_A1 exceeds the threshold when POT_A1 is 127.19 °C. This shows that the anomaly detection method proposed in this study can effectively detect early anomalies of equipment. Using the MD to characterize the residual of the overall model can reduce the false alarm rate compared to using a single variable threshold. Therefore, in the monitoring process, the OR is used to detect the abnormal state of the equipment, and the single variable threshold is used to analyze the cause of the abnormality.

## 4. Conclusions and Future Work

A general anomaly detection framework based on LSTM-AE neural networks is proposed for early detection of abnormalities in power plants. The LSTM-AE neural network-based NBM considers the spatial and temporal correlation of the related operating variables and realizes the simultaneous monitoring of multiple variables. In the case study, hyperparameters of the LSTM-AE model, including the hidden layer unit and look-back time step, were meticulously optimized to obtain a more accurate reconstruction and better generalization. The comparative analysis between the LSTM-AE and PCA-NARX models illustrates that the LSTM-AE model has better performance. The average RMSEs of the training and test datasets are 0.026 and 0.035 °C, respectively. The corresponding average MAPEs are within 0.027%. Based on the similarity analysis between the NBM output value distribution and the corresponding measured value distribution, the MD is used to describe the overall residual of the model. The reasonable residual range of the overall model and each variable were obtained using KDE with a 99% confidence interval. The abnormal operation case shows that the proposed framework detected the abnormality when the variable value was 132.23 °C, however, the conventional fixed threshold is 180 °C. The residual of the overall model is used to detect the abnormal state, and the residual of the single variable is used to analyze the cause of the abnormality. In summary, the proposed framework could be used for the early detection of abnormalities in real-time, which is of great significance to condition-based maintenance in power plants. In future work, an abnormal dataset will be needed to further verify this framework, especially for cases in which multiple variables are simultaneously abnormal. Moreover, the accurate extraction of the normal behavior operation dataset also requires further research.

## Figures and Tables

**Table 1:** The comparison between the LSTM-AE and AE.

	RMSE on Training Dataset	RMSE on Test Dataset	MAPE on Training Dataset	MAPE on Test Dataset
**AE**	0.111	0.191	0.172	0.172
**LSTM-AE**	0.026	0.035	0.027	0.027

**Table 2:** The comparison between LSTM-AE and PCA-NARX.

	RMSE on Training Dataset	RMSE on Test Dataset	MAPE on Training Dataset	MAPE on Test Dataset
**LSTM-AE**	0.026	0.035	0.027	0.027
**PCA-NARX**	0.044	0.044	0.032	0.032
**AE**	0.111	0.191	0.172	0.172

**Table 3:** Reasonable residual ranges of the overall NBM and each variable.

	OR	LOT	POT_A1	POA_A2	MNDT	MDT	MBT_1	MBT_2	MBT_3
***R_lower***	---	−0.048	−0.228	−0.315	−0.041	−0.027	−0.063	−0.029	−0.037
***R_upper***	0.325	0.084	0.341	0.374	0.037	0.066	0.042	0.095	0.064
